# Description of Emergency Medical Services, treatment of cardiac arrest patients and cardiac arrest registries in Europe

**DOI:** 10.1186/s13049-020-00798-7

**Published:** 2020-10-19

**Authors:** Ingvild B. M. Tjelmeland, Siobhan Masterson, Johan Herlitz, Jan Wnent, Leo Bossaert, Fernando Rosell-Ortiz, Kristin Alm-Kruse, Berthold Bein, Gisela Lilja, Jan-Thorsten Gräsner, on behalf of GL2020 Epidemiology group and participating countries, Jan-Thorsten Gräsner, Berthold Bein, Leo Bossaert, Bernd W. Böttiger, Johan Herlitz, Rolf Lefering, Gisela Lilja, Siobhan Masterson, Fernando Rosell-Ortiz, Gavin D. Perkins, Jan Wnent, Sule Akin, Hajriz Alihodzic, Valentine Baert, Marieke Blom, Scott Booth, Roman Burkart, Dave Bywater, Drilon Kamishi, Michael Baubin, Alexei Birkun, Grzegorz Cebula, Diana Cimpoiesu, Giordimaina Christopher, Carlo Clarens, Vitor Hugo Gouveia Correia, Hlynur Höskuldsson, Marios Ioannides, Asta Krikscionaitiene, Stephanie Leckey, Freddy Lippert, Andrej Markota, Pierre Mols, Eniko Nagy, Nikolaos Nikolao, Fernando Rosell-Ortiz, Violetta Raffay, Ari Salo, Federico Semeraro, Stefan Trenkler, Anatolij Truhlář, Martin Quinn

**Affiliations:** 1 Institute for Emergency Medicine, University-Hospital Schleswig-Holstein, Arnold-Heller-Str. 3, 24105 Kiel, Germany; 2 Division of Prehospital Services, Oslo University Hospital, Oslo, Norway; 3 Faculty of Medicine, Institute of Clinical Medicine, University of Oslo, Oslo, Norway; 4 The National Ambulance Service Ireland and the National University of Ireland Galway (on behalf of the Out-of-Hospital Cardiac Arrest Register (OHCAR)), Galway, Ireland; 5 PreHospen - Centre for Prehospital Research, Faculty of Caring Science, Work-Life and Social Welfare, University of Borås, Borås, Sweden; 6 European Resuscitation Council, Niel, Belgium; 7 Department of Anesthesiology and Intensive Care Medicine, University-Hospital Schleswig-Holstein, Kiel, Germany; 8 School of Medicine, University of Namibia, Windhoek, Namibia; 9 University of Antwerp, Antwerp, Belgium; 10 Servicio de Urgencias y Emergencias 061 de La Rioja, Logroño, Spain; 11 Department of Research & Development, Division of Emergencies and Critical Care, Oslo University Hospital, Oslo, Norway; 12 Anaesthesiology and Intensive Care Medicine, Asklepios Hospital St. Georg, Hamburg, Germany; 13 Faculty of Medicine, Semmelweis University, Hamburg, Germany; 14 Department of Clinical Sciences Lund, Neurology, Lund University, Skåne University Hospital, Lund, Sweden

**Keywords:** Emergency Medical Services (EMS), Out-of-hospital cardiac arrest, Cardiac arrest registries, Dispatch, Epidemiology of cardiac arrest

## Abstract

**Background:**

Variation in the incidence, survival rate and factors associated with survival after cardiac arrest in Europe is reported. Some studies have tried to fill the knowledge gap regarding the epidemiology of out-of-hospital cardiac arrest in Europe but were unable to identify reasons for the reported differences. Therefore, the purpose of this study was to describe European Emergency Medical Systems, particularly from the perspective of country and ambulance service characteristics, cardiac arrest identification, dispatch, treatment, and monitoring.

**Methods:**

An online questionnaire with 51 questions about ambulance and dispatch characteristics, on-scene management of cardiac arrest and the availability and dataset in cardiac arrest registries, was sent to all national coordinators who participated in the European Registry of Cardiac Arrest studies. In addition, individual invitations were sent to the remaining European countries.

**Results:**

Participants from 28 European countries responded to the questionnaire. Results were combined with official information on population density. Overall, the number of Emergency Medical Service missions, level of training of personnel, availability of Helicopter Emergency Medical Services and the involvement of first responders varied across and within countries. There were similarities in team training, availability of key resuscitation equipment and permission for ongoing performance of cardiopulmonary resuscitation during transported. The quality of reporting to cardiac arrest registries varied, as well as the data availability in the registries.

**Conclusions:**

Throughout Europe there are important differences in Emergency Medical Service systems and the response to out-of-hospital cardiac arrest. Explaining these differences is complicated due to significant variation in how variables are reported to and used in registries.

## Background

Epidemiology of cardiac arrest and the systems that care for out-of-hospital cardiac arrest (OHCA) patients have been described in many studies. Regional and inter-country variation in survival is a consistent finding in epidemiological studies. In a study from the Resuscitation Outcomes Consortium (ROC), variation between sites was 4.7 to 20% [1]. In 2015, across the seven ambulance services that contribute to the Australian and New Zealand Resuscitation Outcomes Consortium, survival ranged from 9 to 17% [2]. Similarly, from 2009 to 2012, the Pan Asian Resuscitation Outcomes Study (PAROS) observed survival ranging from 0.5 to 8.5% across seven countries [3]. In Europe, the European Registry of Cardiac Arrests (EuReCa) studies showed that between-country OHCA survival ranged from 1.1 to 31% over a 1-month period in 2014 [4], and from 0 to 18% over a 3-month period in 2017 [5].

Identifying the factors that contribute to this variation in OHCA survival is important. The consensus-based Utstein template provides a dataset of patient-level variables associated with survival [6]. Some factors that influence survival are well known i.e. witnessed collapse, bystander cardiopulmonary resuscitation, initial shockable cardiac arrest rhythm and achieved return of spontaneous circulation (ROSC) [7]. It has been estimated in a single city (Toronto) that patient-level Utstein variables accounted for 89% of variability in OHCA survival [8]. In a study from ROC, it was estimated that Utstein variables accounted for 72% of variability across North American sites [9]. However, in a study using international data from 232 Emergency Medical Services (EMS) agencies in 12 countries, the proportion of survival variability accounted for by Utstein variables fell to 51% [10]. The more international the study sites, the greater the variation in interpretation, system, organisation and culture. Hence, the less variation that is explained by patient-level Utstein variables.

Variability in EMS organisation is a common theme across international cardiac arrest registries and epistries [11–14]. It is likely that differences in EMS systems in Europe account for at least some of the differences in OHCA survival. Other factors that might account for the observed variability in survival after OHCA are differences in the links in the chain of survival e.g. the first link (early call for help) [15], or in the fourth link (post-resuscitation care) [16]. Development of different “first responder systems” may also explain part of the variability in survival [17].

In the last European-wide study, EuReCa TWO, the mean incidence rate of started resuscitations was 56 per 100,000 inhabitants per year, ranging from 27 to 91 per 100,000 inhabitants per year [5]. This wide range in national incidence estimates may have been caused by differences in how key variables were interpreted. However, much of this variation is likely to be attributable to patient and system level differences. To date, there has been no comprehensive description of EMS systems in Europe. Therefore, the objective of this study is to describe European EMS systems, particularly from the perspective of ambulance service characteristics, cardiac arrest identification, dispatch, treatment, and monitoring.

## Methods

A structured questionnaire was developed through a review of published literature on previous international ambulance surveys [11–14, 18] and by consensus among members of the European Resuscitation Council (ERC) Guidelines 2020 Epidemiology Writing Group (Writing Group). The questionnaire was designed to investigate the following five categories: (Additional file 1; EMS survey):

1. Country and EMS baseline characteristics
2. Ambulance Service characteristics
3. Dispatch characteristics
4. On-Scene Management of Out-of-Hospital Cardiac Arrest by the EMS
5. Cardiac Arrest Registries

The survey was piloted with the members of the Writing Group. The questionnaire was shared with participants using the online tool Questback, licensed to Oslo University Hospital. All information is stored on an approved area at Oslo University Hospital.

The survey was distributed between October 2019 and January 2020. All national coordinators of the EuReCa ONE or EuReCa TWO studies were asked to participate (*n* = 31). Representatives from other European countries were invited to participate using the ERC network and the individual networks of the Writing Group (*n* = 3). In total the survey was sent to 34 different countries. Participants were asked to provide information for the entire country.

After completion of the survey, results were returned to each participant, who was asked to validate responses with at least one other national expert. Countries that did not confirm their response were excluded from the survey. In case of inconsistencies or critical missing data, participants were again contacted to maximise data quality. After all the data had been merged into a result section, the tables were again shared with the participants, who then confirmed the results. All participants were asked for consent to be acknowledged in publications and reports. Participants were entitled to withdraw from the study at any time up to submission of the article.

Descriptive analysis of data was carried out using Statistical Package for Social Sciences (SPSS, Inc., IL, USA) version 23. Results are presented as frequencies and proportions.

## Results

### Country information and baseline characteristics

Survey responses from 33 out of 39 (85%) respondents were received. Three responses were excluded as results related to only one region (*n* = 2) or validation of results was not received (*n* = 1). For the United Kingdom, separate answers were received for England, Scotland and Northern Ireland and the answers were merged. A total of 28 countries were included in the analysis.

**Fig. 1:**
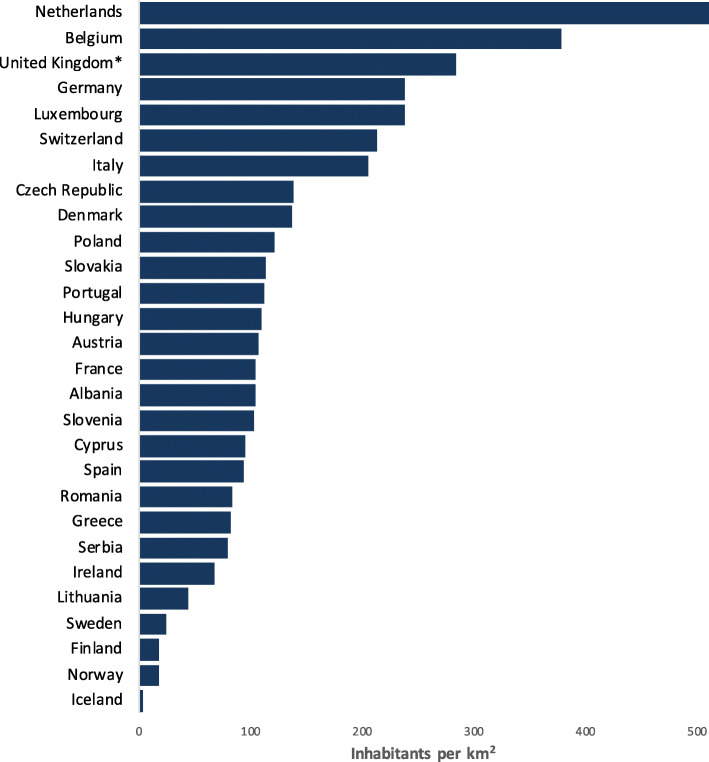
Population per km^2^ in Countries Surveyed. Legend: Population density calculated per km^2^ in relation to total population of the country. The numbers are from the official webpage of the European Union, Europa.eu. * Unite Kingdom excluding Wales

For participating countries, national populations varied from 375,000 in Iceland to over 83 million in Germany [19]. Population density ranged from 3.6 to almost 510 population/km^2^ (Fig. 1). Data on the number of EMS missions per 1000 inhabitants per year were available for 19 countries and varied from 12 in France to 268 in Lithuania. In 75% of countries the EMS was described as publicly funded. Germany had the greatest number of hospitals per million inhabitants while Finland had the lowest (23 vs 3.6 respectively). Only Albania and Cyprus did not operate bypass protocols to bring patients directly to a Percutaneous Coronary Intervention (PCI)-capable hospital. The majority of respondents (*n* = 25) also reported that there were “Cardiac Arrest” hospitals in their country i.e. hospitals capable of providing all of the following post-resuscitation interventions: 24/7 primary PCI, targeted temperature management and neuro-prognostication. Data on the median response times for urban and rural areas is presented in Table 1.

**Table 1:** Baseline characteristics of participating countries

Country	Population	EMS Missions per 1000 inhabitants	Public or Private EMS funding?	Hospitals per million population	“Cardiac Arrest” hospitals	PCI bypass protocol	Rural areas - median ambulance response time < 10 min?	Urban - median ambulance response time < 10 min?
**Albania**	2,862,400		Public		Some areas	No	Some areas	Some areas
**Austria**	8,858,800		Public	10.4	All areas	Yes	Some areas	Some areas
**Belgium**	11,467,900	77.0	Public and private	10.9	All areas	Yes	Some areas	All areas
**Cyprus**	875,900	62.8	Public	8.0	All areas	No	Some areas	Some areas
**Czech Republic**	10,649,800	103.9	Public		Some areas	Yes	Some areas	All areas
**Denmark**	5,806,100	68.9	Public	3.8	Some areas	Yes	Some areas	All areas
**Finland**	5,517,900	140.1	Public	3.6	Some areas	Yes	Some areas	Some areas
**France**	67,028,000	11.9	Public	9.8	Some areas	Yes	No	Some areas
**Germany**	83,019,200	172.2	Public	22.9	Some areas	Yes	Some areas	Some areas
**Greece**	10,722,300		Public		None	Yes	Some areas	Some areas
**Hungary**	9,772,800		Public	5.9	Some areas	Yes	Some areas	Some areas
**Iceland**	357,000	117.6	Public	14.0	Some areas	Yes	Some areas	All areas
**Ireland**	4,693,460	95.9	Public	6.2	Some areas	Yes	No	Some areas
**Italy**	60,359,500		Public	4.7	Some areas	Yes	Some areas	All areas
**Lithuania**	2,794,200	268.4	Public and private	14.3	None	Yes	No	No
**Luxembourg**	613,900	72.5	Public	4.9	Some areas	Yes	Some areas	All areas
**Netherlands**	17,282,200	57.6	Public and private	5.0	All areas	Yes	Some areas	Some areas
**Norway**	5,323,933	136.2	Public	9.4	Some areas	Yes	Some areas	All areas
**Poland**	37,972,800		Public	8.6	Some areas	Yes	No	All areas
**Portugal**	10,276,600	114.2	Public	4.3	Some areas	Yes	No	Some areas
**Romania**	19,401,700	172.1	Public and private	6.7	Some areas	Yes	Some areas	Some areas
**Serbia**	6,963,800	43.7	Public	7.0	Some areas	Yes	No	Some areas
**Slovakia**	5,450,400	26.6	Public and private	13.8	None	Yes	Some areas	Some areas
**Slovenia**	2,080,900		Public	5.3	All areas	Yes	Some areas	Some areas
**Spain**	46,934,600		Public	4.9	Some areas	Yes	No	Some areas
**Sweden**	10,230,200	97.7	Public and private	7.2	Some areas	Yes	Some areas	Some areas
**Switzerland**	8,542,300	58.5	Public and private	11.9	Some areas	Yes	Some areas	All areas
**United Kingdom**^a^	63,298,819		Public		Some areas	Yes	Some areas	Some areas

For country population official numbers from EU were used. (eurpoa.eu)

*Abbreviations*: *EMS* Emergency Medical Services, *PCI* Percutaneous Coronary Intervention, “*Cardiac arrest hospitals*” - hospitals capable of providing all of the following post-resuscitation interventions: 24/7 primary PCI, targeted temperature management and neuro-prognostication

^a^United Kingdom excluding Wales

### Ambulance service characteristics

**Fig. 2:**
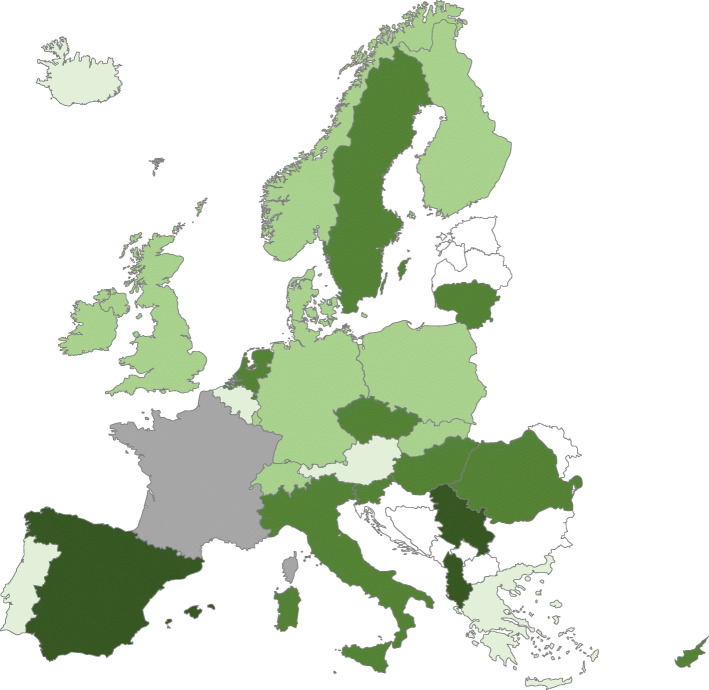
Occupation of the majority of employees in the EMS. Legend: The darkest colour indicates countries where the majority of employees are medical doctors, the second darkest colour emergency nurses/nurses, light green indicates paramedics, very light indicates emergency medical technicians and grey is other. White colour indicates the country did not participate in the survey

In 15 countries, the majority of EMS personnel were reported as paramedics or Emergency Medical Technicians (EMTs) with at least 2 years of specialist training (Fig. 2). It was reported that all ambulance personnel were trained in Advanced Life Support (ALS) in 19 countries (i.e. at least ERC ALS level or similar), and at least some were trained in the remaining countries. In 12 countries, non-physician ambulance personnel were allowed to perform ALS procedures in the absence of a physician (see Table 2).

**Table 2:** Ambulance service characteristics – training and occupation of EMS ambulance personnel

Country	Ambulance personnel ALS trained?	What is the occupation of the majority of EMS personnel?	Do physicians provide patient care as part of EMS?	Can ALS trained ambulance personnel perform the following interventions without a physician present on scene?			
				Secure airways with supraglottic or endotracheal tubes	Intravenous or intraosseous drug therapy	Manual defibrillation	Semi-automatic defibrillation
**Albania**	Some	Emergency physician	Sometimes	No	Yes	Yes	Yes
**Austria**	All	EMT	Routinely	Yes	Yes	No	Yes
**Belgium**	Some	EMT	Routinely	No	No	No	Yes
**Cyprus**	All	Emergency nurse / nurse	No	Yes	Yes	Yes	Yes
**Czech Republic**	Some	Emergency nurse / nurse	Routinely	Yes	Yes	Yes	Yes
**Denmark**	All	Paramedic	Routinely	Yes	Yes	Yes	Yes
**Finland**	Some	Paramedic	Routinely	Yes	Yes	Yes	Yes
**France**	Some	Other	Routinely	Yes	Yes	Yes	Yes
**Germany**	All	Paramedic	Routinely	Yes	Yes	Yes	Yes
**Greece**	Some	EMT	Sometimes	No	No	No	No
**Hungary**	Some	Emergency nurse / nurse	Routinely	Yes	Yes	Yes	Yes
**Iceland**	Some	EMT	Sometimes	Yes	Yes	Yes	Yes
**Ireland**	All	Paramedic	Sometimes	Yes	Yes	Yes	Yes
**Italy**	Some	Emergency nurse / nurse	Routinely	Yes	Yes	No	Yes
**Lithuania**	Some	Emergency nurse / nurse	Sometimes	Yes	Yes	Yes	Yes
**Luxembourg**	Some	Paramedic	Routinely	Yes	No	No	Yes
**Netherlands**	All	Emergency nurse / nurse	Sometimes	Yes	Yes	Yes	Yes
**Norway**	All	Paramedic	Sometimes	Yes	Yes	Yes	Yes
**Poland**	All	Paramedic	Sometimes	Yes	Yes	Yes	Yes
**Portugal**	Some	EMT	Routinely	Yes	Yes	No	Yes
**Romania**	Some	Emergency nurse / nurse	Sometimes	Yes	Yes	Yes	Yes
**Serbia**	Some	Emergency physician	Routinely	No	No	No	No
**Slovakia**	All	Paramedic	Routinely	Yes	Yes	Yes	Yes
**Slovenia**	All	Emergency nurse / nurse	Routinely	Yes	Yes	Yes	Yes
**Spain**	All	Emergency physician	Routinely	No	Yes	Yes	Yes
**Sweden**	All	Emergency nurse / nurse	Sometimes	Yes	Yes	Yes	Yes
**Switzerland**	All	Paramedic	Routinely	Yes	Yes	Yes	Yes
**United Kingdom**^a^	Some	Paramedic	Sometimes	Yes	Yes	Yes	Yes

The answers are for the entire country which means that the answers “Some” and “Sometimes” indicate this is not implemented in all EMS services in the entire country

*Abbreviations*: *EMS* Emergency Medical Services, *EMT* Emergency Medical Technician, ALS – Advanced Life Support

^a^United Kingdom excluding Wales

**Fig. 3:**
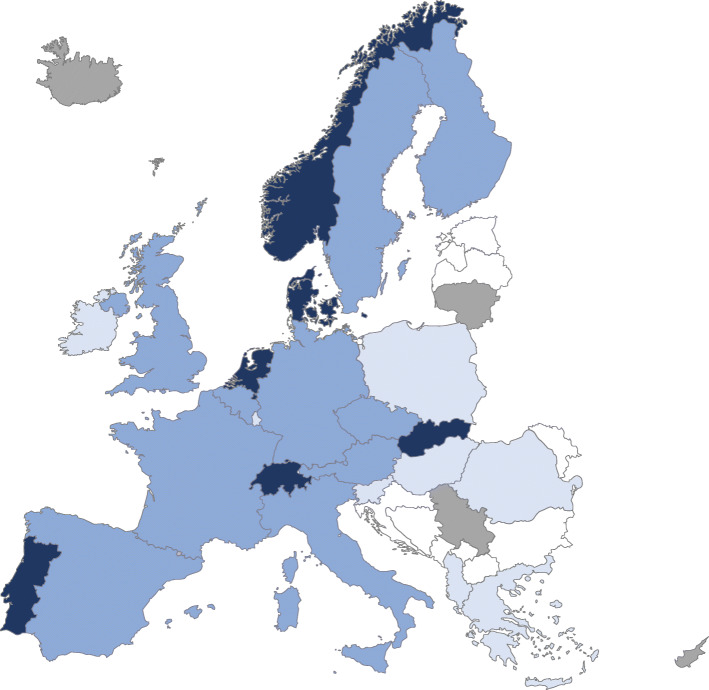
Availability of Helicopter Emergency Medical Services. Legend: Dark blue indicates HEMS 24/7 in all of the country, medium blue indicates HEMS 24/7 in some areas, light blue indicates HEMS but not 24/7 and grey indicates no HEMS. White colour indicates the country did not participate in the survey

A Helicopter EMS (HEMS) was available in 24 countries. Cyprus, Iceland, Lithuania and Serbia reported that they did not operate HEMS. Denmark, the Netherlands, Norway, Portugal, Slovakia, and Switzerland reported having 24/7 HEMS availability in all areas (Fig. 3).

In 18 countries there were established first responder systems (where volunteers were alerted to OHCA by the dispatch centre) in some or all areas. Countries that were reported not to have first responder systems were Albania, Belgium, Cyprus, Finland, Greece, Poland, Portugal, Serbia and Slovakia (no information received about Italy). In eight countries, volunteers were reported to staff ambulances in the EMS (i.e. Austria, Belgium, Germany, Hungary, Italy, Luxembourg, Portugal and Romania).

### Dispatch characteristics

The number of dispatch centres per million population ranged from 3.3 in Germany to 0.3 in Albania. Dispatch centres were part of the EMS in 18 countries, while four countries had some dispatch centres as part of the EMS. It was reported that all countries, with the exception of Serbia, operated a standardised dispatch protocol in all or some of the country (no data available for Greece). Dispatch-assisted Cardio Pulmonary Resuscitation (DA-CPR) instructions were offered in all countries except Greece, but a standardised DA-CPR protocol was not reported to be in use in Poland and Serbia. The type of DA-CPR that was offered was compressions only in ten countries, situation dependent in 15 countries and full CPR with compression and ventilation in two countries. Dynamic deployment, meaning sending the nearest available ambulance/EMS resource, was reported in all countries. However, this was only in some areas of Germany, Romania, Serbia, Slovenia, Spain, Switzerland and the United Kingdom. In 21 countries there were registries of publicly available Automated External Defibrillators (AED) in at least some areas (see Table 3).

**Table 3:** Dispatch characteristics

Country	Dispatch centres per million inhabit-ants	Are the dispatch centres part of EMS?	Standar-dised dispatch protocol used in dispatch centres?	Dispatch-assisted CPR offered?	Type of dispatch-assisted CPR offered	Standard protocol for dispatch-assisted CPR used?	Dynamic deploy-ment used?	Registries of publicly available AED?	AED registries available in dispatch centres?
**Albania**	0.3		All	Yes	Compressions only	Yes	Yes	No	
**Austria**	1	Some	Some	Yes	Situation dependent	Some areas	Yes	All areas	All
**Belgium**	0.9	None	All	Yes	Full CPR	Yes	Yes	No	
**Cyprus**	1.1	All	All	Yes	Compressions only	Yes	Yes	No	
**Czech Republic**	1.3	All	Some	Yes	Situation dependent	Yes	Yes	All areas	All
**Denmark**	0.9	All	All	Yes	Situation dependent	Yes	Yes	All areas	All
**Finland**	1.1	None	All	Yes	Situation dependent	Yes	Yes	All areas	No
**France**	1.5	Some	All	Yes	Situation dependent	Yes	Yes	Some areas	Some
**Germany**	3.3	All	Some	Some areas	Situation dependent	Some areas	Some areas	Some areas	Some
**Greece**	1	All		No		No	Yes	No	
**Hungary**	0.7	All	All	Yes	Compressions only	Yes	Yes	All areas	All
**Iceland**	2.8	None	All	Yes	Full CPR	Yes	Yes	No	
**Ireland**	0.4	All	All	Yes	Compressions only	Yes	Yes	Some areas	Some
**Italy**	1.2	Some	All	Yes	Situation dependent	Yes	Yes	Some areas	Some
**Lithuania**	1.8	All	Some	Some areas	Situation dependent	Some areas	Yes	No	
**Luxembourg**	1.6	All	All	Yes	Compressions only	Yes	Yes	All areas	No
**Netherlands**	1	All	All	Yes	Situation dependent	Yes	Yes	All areas	Some
**Norway**	3	All	All	Yes	Situation dependent	Yes	Yes	All areas	All
**Poland**	0.4	None	All	Some areas	Situation dependent	No	Yes	Some areas	Some
**Portugal**	0.6	Some	Some	Yes	Compressions only	Yes	Yes	Some areas	Some
**Romania**	2.1	All	All	Yes	Compressions only	Some areas	Some areas	Some areas	Some
**Serbia**	0.6	All	No	Some areas	Situation dependent	No	Some areas	No	
**Slovakia**	1.5	None	All	Yes	Compressions only	Yes	Yes	Some areas	Some
**Slovenia**	1	All	All	Yes	Situation dependent	Yes	Some areas	Some areas	All
**Spain**	0.7	All	Some	Yes	Compressions only	Yes	Some areas	Some areas	Some
**Sweden**	1.4	All	All	Yes	Situation dependent	Yes	Yes	All areas	All
**Switzerland**	1.8	All	All	Yes	Compressions only	Yes	Some areas	Some areas	Some
**United Kingdom**^a^	0.4	All	All	Yes	Situation dependent	Yes	Some areas	Some areas	Some

The answers are for the entire country which means that the answers “Some” and “Some areas” indicate this is not implemented in all dispatch centres in the entire country. Empty field means no information was given for that specific question

*Abbreviations*: *EMS* Emergency Medical Services, *AED* Automated External Defibrillator

^a^United Kingdom excluding Wales

### On-scene management of out-of-hospital cardiac arrest by Emergency Medical Services

Team training in CPR involving all EMS personnel was reported in 27 countries, but only 12 countries had this in all areas. Defibrillators were available in all EMS vehicles dispatched to OHCA, with the exception of Albania. Real-time CPR performance data was collected for feedback and debriefing purposes in 17 countries, but used in all areas in Cyprus only. Mechanical CPR was used in 24 countries, and transport with ongoing CPR was permitted in all countries except Luxembourg. However, 23 respondents described specific circumstances in which transport with ongoing CPR may be considered. Eighteen countries were reported to use thrombolysis in OHCA. Availability of more advanced resuscitation interventions on-scene was limited, with extracorporeal membrane oxygenation (ECMO) reported as being used in five countries (France, Germany, Italy, Poland and Portugal), and resuscitative endovascular balloon occlusion of the aorta (REBOA) reported in three countries only (Germany, Italy, and Norway) On-scene management of OHCA is presented in Table 4.

**Table 4:** On scene management of out-of-hospital cardiac arrest by emergency medical personnel in the participating countries

Country	Is there team training in CPR involving all EMS personnel?	Mechanical CPR used?	Real-time CPR performance data collected for feedback?	Transport with ongoing CPR performed?	Defibrillators available in EMS vehicles dispatched for cardiac arrest?	Thrombolysis used in OHCA?
**Albania**	Some areas	No		Yes	Sometimes	No
**Austria**	Some areas	Some areas		Yes	Always	Some areas
**Belgium**	Some areas	Some areas	Some areas	Yes	Always	Some areas
**Cyprus**	Yes	All areas	Yes	Yes	Always	No
**Czech Republic**	Some areas	Some areas	Some areas	Yes	Always	Some areas
**Denmark**	Some areas	Some areas	Some areas	Yes	Always	Yes
**Finland**	Some areas	Some areas	Some areas	Yes	Always	Some areas
**France**	Yes	All areas		Yes	Always	Some areas
**Germany**	Yes	Some areas	Some areas	Yes	Always	Yes
**Greece**	Yes	No	Some areas	Yes	Always	No
**Hungary**	Some areas	Some areas	No	Yes	Always	No
**Iceland**	Yes	Some areas	Some areas	Yes	Always	No
**Ireland**	Yes	All areas	No	Yes	Always	No
**Italy**	Some areas	Some areas	Some areas	Yes	Always	Some areas
**Lithuania**	Some areas	Some areas	No	Yes	Always	No
**Netherlands**	Yes	Some areas	Some areas	Yes	Always	Yes
**Norway**	Some areas	Some areas	No	Yes	Always	Some areas
**Poland**	Some areas	Some areas	Some areas	Yes	Always	No
**Portugal**	Yes	No	No	Yes	Always	Yes
**Romania**	Yes	Some areas	Some areas	Yes	Always	Some areas
**Serbia**	Yes	Some areas	No	Yes	Always	Yes
**Luxembourg**	No	No	No	No	Always	Some areas
**Slovakia**	Some areas	Some areas	Some areas	Yes	Always	Some areas
**Slovenia**	Yes	Some areas	Some areas	Yes	Always	Yes
**Spain**	Some areas	Some areas	Some areas	Yes	Always	Some areas
**Sweden**	Yes	Some areas	No	Yes	Always	No
**Switzerland**	Some areas	Some areas	Some areas	Yes	Always	
**United Kingdom**^a^	Some areas	Some areas	Some areas	Yes	Always	Some areas

On scene management of out-of-hospital cardiac arrest by emergency medical personnel, including information on team training for all involved in the treatment of cardiac arrest paitents

*Abbreviations*: *EMS* Emergency Medical Services, *OHCA* Out-of-Hospital Cardiac Arrest

^a^United Kingdom excluding Wales

### Cardiac arrest registries

**Fig. 4:**
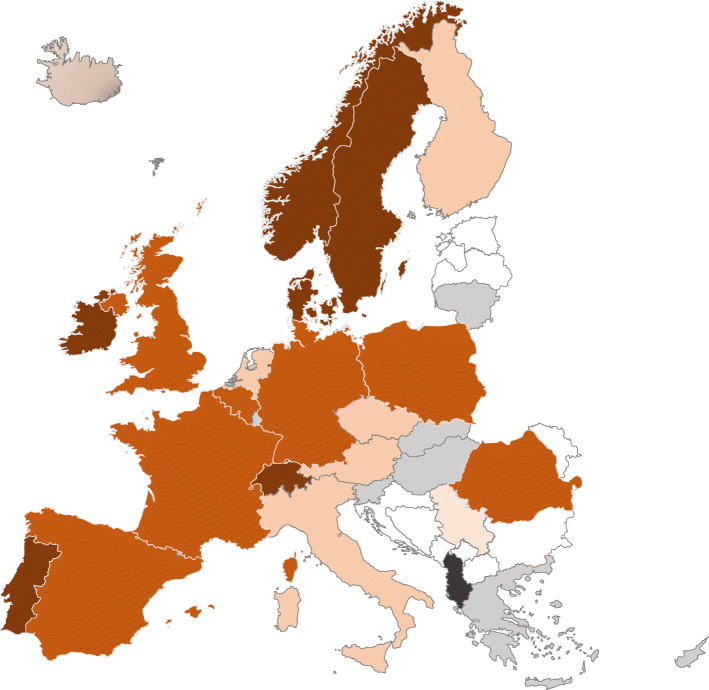
Out-of-hospital cardiac arrest registries. Legend: The darkest colour indicates a national registry covering all of the country, the second darkest colour indicates a national registry covering parts of the country, medium orange indicates several local registries, light with grey indicates one local registry, grey indicates no local registries and black is unknown. White colour indicates the country did not participate in the survey

Six countries reported having an OHCA registry with full population coverage (Denmark, Ireland, Norway, Portugal, Sweden and Switzerland), while partial coverage was described for 14 countries. Seven countries were reported not to have a registry (data not available for Albania) (Fig. 4). Of the 20 countries reported to have full or partial registries, information on the types of outcome data collected was limited, and only Italy reported collecting all outcome variable types, albeit only in some areas of the country (see Table 5). Information in registries about the patients’ neurological status at discharge was available in 13 registries, but follow-up after discharge and the patients reported quality of life was limited to data collection in some areas of seven countries.

**Table 5:** Cardiac arrest registry coverage and outcome variables collected

Country	Out-of-hospital cardiac arrest registry?	Any ROSC?	Sustained ROSC?	Status on arrival at hospital arrival?	Survival to hospital discharge?	Survival to 30 days?	Survival to one year?	CPC at discharge?	CPC at 3 to 6 months?	CPC at 1 year?	QoL?	Self-defined QoL?
**Albania**												
**Austria**	Several local registries	Some areas	Some areas	Some areas	Some areas	Some areas		Some areas				
**Belgium**	National registry, partial coverage	All areas	All areas	All areas	All areas	All areas		All areas				All areas
**Cyprus**	No											
**Czech Republic**	Several local registries	Some areas		Some areas	Some areas	Some areas		Some areas				
**Denmark**	Full national coverage	All areas		All areas		All areas	All areas					
**Finland**	Several local registries	Some areas	Some areas	Some areas	Some areas		Some areas	Some areas				
**France**	National registry, partial coverage	All areas		All areas	All areas	All areas		All areas	All areas			
**Germany**	National registry, partial coverage	All areas	All areas	All areas	Some areas	Some areas	Some areas	Some areas		Some areas		Some areas
**Greece**	No											
**Hungary**	No											
**Iceland**	Single local registry	Some areas	Some areas	Some areas								
**Ireland**	Full national coverage	All areas	All areas	All areas	All areas			All areas				
**Italy**	Several local registries	Some areas	Some areas	Some areas	Some areas	Some areas	Some areas	Some areas	Some areas	Some areas	Some areas	Some areas
**Lithuania**	No											
**Luxembourg**	No											
**Netherlands**	Several local registries	Some areas	All areas	All areas	All areas	All areas	All areas	All areas				
**Norway**	Full national coverage	All areas	All areas	All areas	All areas	All areas		All areas				
**Poland**	National registry, partial coverage	Some areas	Some areas	Some areas	Some areas	Some areas	Some areas					
**Portugal**	Full national coverage	All areas	All areas	All areas								
**Romania**	National registry, partial coverage	Some areas	Some areas	Some areas	Some areas							
**Serbia**	Several local registries	All areas	All areas	All areas	All areas	All areas						
**Slovakia**	No											
**Slovenia**	No											
**Spain**	National registry, partial coverage	All areas	All areas	All areas	Some areas	Some areas	Some areas	Some areas	Some areas	Some areas		
**Sweden**	Full national coverage	All areas	All areas	All areas	Some areas	All areas		Some areas	Some areas		Some areas	Some areas
**Switzerland**	Full national coverage	All areas	All areas	All areas	Some areas	Some areas	Some areas	Some areas		Some areas		
**United Kingdom**^a^	National registry, partial coverage	All areas	All areas	All areas	All areas	All areas						

*Abbreviations*: *ROSC* Return of Spontanious Circulation, *CPC* Cerebral Performance Category, *QoL* Quality of Life

^a^United Kingdom excluding Wales

## Discussion

To the best of our knowledge this survey, covering 28 countries, provides the most comprehensive overview of EMS systems in Europe to date, particularly with regard to out-of-hospital cardiac arrest. The survey uncovers variations in service characteristics that are not fully explained in relation to total population, population density or geography. Our findings of substantial variation follow the pattern observed when EMS systems have been compared elsewhere [11–14].

There are some baseline characteristics shared by European countries in that the majority have publicly funded EMS systems and hospital bypass protocols for OHCA. However, our results suggest that while total population explained some proportion of variation, there remains large differences in fundamental measures of EMS activity such as EMS missions per 1000 inhabitants, and the capacity to respond to patients in a median of 10 min. Similarly, most countries were reported to have hospitals capable of providing post-resuscitation care as recommended in the ERC resuscitation guidelines 2015 [20], but there were vast differences in the number of hospitals with 24/7 emergency departments per 1 million inhabitants.

Our survey has shown differences in the types of personnel employed as part of the EMS and in the levels and types of interventions that EMS personnel are allowed to carry out independent of physician supervision. Previous studies have demonstrated how differences in EMS organisation may contribute to variation in OHCA survival. A prospective study showed that higher qualification and greater training experience of ambulance personnel contributed to higher OHCA survival across the four participating EMS agencies [21]. Across the ten ROC sites, differences in EMS practice with regard to initiation of resuscitation and transport was found to contribute to variation in OHCA survival [22], and EMS agencies with the highest survival rates more often had: treatment from more than six EMS personnel; a shorter EMS call-response interval; more advanced airway attempts; and treatment from an advanced-basic life support tiered system [23].

Cardiac arrest is highly time-sensitive and after 10 min with no CPR or defibrillation, the chances of survival are slim. Median response times for urban areas in Europe of under 10 min were achieved in only 32% of the countries. It is therefore encouraging that our survey has reported that at least 18 European countries have established first responder systems. However, another recent European survey described that many different kinds of first responder systems are used, and also highlighted that regions within countries had different approaches [24]. The introduction of first responder systems is positive, but further layers of difference now need to be considered when explaining variation in outcomes. Of the countries included in our survey, 67% had all dispatch centres as part of the EMS while 15% had some dispatch centres as part of the EMS. The size of the country or the total population did not seem to be the determining factor in the number of dispatch centres. For example, despite differences in population density, Germany and Norway have approximately three dispatch centres per million inhabitants. Similarly, Poland, UK, Ireland and Albania are vastly different in terms of population and land mass, but all have less than 0.5 dispatch centres per million inhabitants. It is important to note that the vast majority of countries reported the use of standardised dispatch protocols and dispatch-assisted CPR instructions. While there was variation in the type of instructions offered, evidence on the type of dispatch-assisted CPR instructions that should be offered is still building [25, 26]. There is increasing evidence of the value of publicly accessible AEDs [27, 28], therefore it was encouraging that responses indicated availability of AED registries in 21 countries. Most importantly, the majority of these registries were available in dispatch centres.

Time-to-shock is a critical determinant of survival [29], therefore the availability of defibrillators in EMS vehicles dispatched for cardiac arrest was a positive finding. Evidence on the value of mechanical CPR remains equivocal [30, 31], which may explain why mechanical CPR was reported to be available in all areas in only three countries. Availability of more advanced prehospital resuscitation interventions was limited, which may also be explained by the current limited evidence to support widespread adoption of these practices. It is of note that most countries permitted transport with ongoing CPR. However, most respondents described very specific circumstances for this practice.

In 2012 the European Parliament published a declaration recommending that all member states adopt common programs for implementing AEDs in public places and training of lay people, adjusting of legislation in order to facilitate CPR and defibrillation by non-medical persons, and organisation of systematic data collection on cardiac arrest for feedback and quality management [32]. Registry data collection in itself is not a guarantee for improved survival, but if core data variables are not available, routine monitoring and surveillance of OHCA outcomes may be difficult. In our survey only six countries reported having a registry with full population coverage and 14 countries reported having partial population coverage. In these registries, availability of core outcome variables including ROSC was limited. The establishment of cardiac arrest registries in 20 out of 28 countries is promising, but renewed focus is needed to encourage countries to ensure that outcome data is a core component of data collection, as outcome data is essential to compare results and benchmark against the countries that have achieved high survival rates.

There are a number of limitations to this survey. Firstly, the questionnaire was distributed via an established network, primarily developed for conducting the EuReCa ONE and TWO studies. This network has a specific interest in and responsibility for OHCA management and data collection. While there is a risk of selection bias, it is assumed that respondents have a prior knowledge of the EMS systems in their countries. Additionally, respondents were required to validate their answers with another national expert. Secondly, respondents were required to provide answers about their entire country therefore differences in EMS systems within countries were not the focus of this survey. However, respondents were given the option to answer ‘sometimes’ or ‘in some areas’ where appropriate. Finally, the survey was conducted in English but this is not the primary spoken language for most countries that participated. It is therefore possible that there may have been differences in interpretation of questions by different respondents.

This survey has described some of the differences in the EMS systems in Europe and have raised a number of new research questions. In future, research surveys should be set up to look for correlations or associations between variables, and linking the results to outcome after out-of-hospital cardiac arrest and survival after trauma. In addition, future research on EMS systems in Europe should consider using the WHO emergency care system assessment tool.

## Conclusion

Throughout Europe there are significant differences in EMS systems and the response to OHCA. Even for interventions that have been shown to have an effect on survival, implementation across Europe varies. While the impact of EMS system differences is not fully understood, having documented these differences provides the opportunity to adjust for the differences when looking at incidence and survival after OHCA.

## Supplementary information

**Additional file 1.** EMS survey.

## Data Availability

The individual responses to the survey are available from the corresponding author on reasonable request. Consent from all involved participants will be sought before sharing.

## Abbreviations

OHCA: Out-of-hospital cardiac arrest
ROC: Resuscitation Outcomes Consortium
Aus-ROC: Australian and New Zealand Resuscitation Outcomes Consortium
PAROS: Pan Asian Resuscitation Outcomes Study
EuReCa: European Registry of Cardiac Arrests
ROSC: Return of spontaneous circulation
EMS: Emergency Medical Services
ERC: European Resuscitation Council
Writing Group: Guidelines 2020 Epidemiology Writing Group
PCI: Percutaneous Coronary Intervention
EMTs: Emergency medical technicians
ALS: Advanced life support
HEMS: Helicopter EMS
DA-CPR: Dispatch-assisted Cardio Pulmonary Resuscitation
AED: Automated External Defibrillators
ECMO: Extracorporeal membrane oxygenation
REBOA: Resuscitative endovascular balloon occlusion of the aorta
