# Supplementary material for: Description of Emergency Medical Services, treatment of cardiac arrest patients and cardiac arrest registries in Europe
Source: Scand J Trauma Resusc Emerg Med. 2020 Oct 19;28:103. doi: 10.1186/s13049-020-00798-7 (PMC7569761; doi:10.1186/s13049-020-00798-7)
Supplement: Supplementary file 1 — Additional file 1. EMS survey. [file 13049_2020_798_MOESM1_ESM.pdf]

**EPIDEMIOLOGY SURVEY OF EMS SYSTEMS IN EUROPE**

**Updated 10th of September 2019**

Baseline Characteristics

1. Country
   - Dropdown all European countries
2. Total number of started EMS missions per year in your country (including missions where EMS was alarmed but later "stood down")
   - Number
   - Unknown
3. How is the EMS funded in your country?
   - Privat
   - Public
   - Both
   - Other – open answer
4. Number of hospitals with a 24/7 emergency department in your country?
   (Definition: an emergency department/acute hospital can receive seriously ill or injured patients on a 24 hour basis )
   - Numbers only
   - Unknown
5. Are specialized Cardiac Arrest Centres for post Resuscitation care available in your country?
   (Definition of cardiac arrest centres: institutions that have access to a 24-h cardiac catheterisation laboratory, targeted temperature management in a critical care facility and prognostication using multimodal approach including delayed clinical examination, neuroelectrophysiological measurements and biomarkers, (ERC Guidelines 2015))
   - Yes, all areas
   - Yes, some areas
   - None
   - Unknown
6. Are there bypass protocols in place which allow the EMS to bring patients directly to a primary PCI-capable hospital?
   - Yes
   - No
   - Unknown
7. Rural areas, median ambulance response time <10 minutes?
   - Yes, all areas
   - Yes, some areas
   - No
   - Unknown
8. Urban areas, median ambulance response time <10 minutes?
   - Yes, all areas
   - Yes, some areas
   - No
   - Unknown

## Ambulance Service Characteristics

9. What is the occupation of the majority of health care providers in the EMS?
    - o Emergency physician
    - o Emergency nurse
    - o Paramedic
    - o Other
    - o Unknown
10. Does your EMS have a standardised report form for all EMS interventions?
    - o Yes, all areas
    - o Yes, some areas
    - o No
    - o Unknown
11. Does your country have a helicopter EMS?
    - o Yes
    - o No
    - o Unknown

If yes in question 11, answer 12 and 13, if no go to 14

12. Is helicopter EMS available 24/7?
    - o Yes, all areas
    - o Yes, some areas
    - o No
    - o Unknown
13. What proportion of your country is covered by the helicopter 24/7?
    - o 0-10
    - o 11-20
    - o 21-30
    - o 31-40
    - o 41-50
    - o 51-60
    - o 61-70
    - o 71-80
    - o 81-90
    - o 91-100

14. Does your country have an established first-responder system (alerted to OHCA by dispatch)? (Definition of first responder: a person who is alerted and directed to the scene by an ambulance dispatch centre to help, but is NOT on-duty EMS personnel)
    - o Yes, all areas/regions
    - o Yes, some areas/regions
    - o No
15. In your ordinary EMS service, do you have volunteers staffing the ambulances?
    - o Yes

- No
- Unknown

16. Level of training  of ambulance personnel in your country
(ALS trained personnel have an ERC ALS course or similar)
    - All are ALS trained
    - Some are ALS trained
    - None are ALS trained
17. Is equipment and expertise available for life-threatening trauma (life, limb) in all emergency ambulances?
    - Yes, all
    - Yes, some
    - None
    - Unknown
18. ALS trained ambulance personnel in our country can do the following at arrival on scene without a doctor present (multiple choice):
    (ALS trained personnel have an ERC ALS Course or similar training
    - Secure airways with supraglottic or endotracheal tubes
    - Intravenous/IO drug therapy
    - Manual defibrillation
    - Semi-automatic defibrillation
    - None of the above
19. Do physicians provide patient care on scene for OHCA patients as part of EMS?
    - Yes, routinely
    - Yes, sometimes
    - No
    - Unknown
20. Are personnel on the first responding ambulance ALS trained?
    - Yes, always
    - Sometimes
    - No
    - Unknown
21. Are personnel on the second responding ambulance ALS trained
    - Yes, always
    - Sometimes
    - No
    - Unknown

## Dispatch Characteristics

22. How many ambulance dispatch centres are in your country?
    - Number
    - Unknown
23. Are the dispatch centres part of your ambulance service?
    - All
    - Some
    - None

- Unknown

24. What is the approximate number of medical emergency calls received for your country in one year?
    - Number only
    - Unknown
25. Is a standardised dispatch protocol used in your country?
    - Yes, in all dispatch centres
    - Yes, in some
    - No
    - Unknown
26. Is ‘dynamic deployment’ used?

Dynamic deployment means sending the nearest available ambulance/EMS resource

- Yes
- In some areas
- No

27. Is dispatch assisted bystander CPR offered?
    - Yes
    - In some areas
    - No
28. Dispatch assisted CPR is:
    - Compressions only
    - Full CPR
    - Situation dependent
    - Not implemented
29. Is a standard protocol for dispatch assisted bystander CPR used?
    - Yes
    - In some areas
    - No

<u>OHCA Resuscitation</u>

30. Is real-time CPR performance data collected for feedback during CPR and/or debrief post-cardiac arrest?
    - Yes
    - In some areas
    - No
    - Unknown
31. Is mechanical CPR used by ambulance personnel?
    - Yes, in all of the country
    - Yes, in some areas
    - No
    - Unknown
32. Is advanced equipment used for resuscitation outside hospital in any part of your country (multiple choice)?
    - ECMO

        - REBOA
        - Other
        - No
        - Unknown

33. Is transport with ongoing CPR performed?
    - Yes
    - Sometimes
    - No
    - Unknown

34. If yes in question 33: Under what circumstances is transport with ongoing CPR performed?
    - Open answer
35. Is thrombolysis used in out-of-hospital cardiac arrest?
    - Yes
    - In some areas
    - No
    - Unknown
36. Is a system for intraosseous (IO) access implemented and routinely used by ambulance personnel?
    - Yes
    - In some areas
    - No
    - Unknown
37. Is a system for intraosseous (IO) access implemented and routinely used by emergency physicians?
    - Yes
    - In some areas
    - No
    - Unknown
38. Is a team training in CPR for all EMS members implemented (ALS- BLS-paramedics, emergency physicians)?
    - Yes
    - In some areas
    - No
    - Unknown
39. Are defibrillators (manual and/or automated external) available in vehicles dispatched for cardiac arrest?
    - Yes, all
    - Yes, some
    - No
    - Unknown
40. Are there registries of publicly available AEDs in your country?
    - Yes, all of the country
    - Yes, parts of the country
    - No

- Unknown

41. If yes, available in the dispatch centre
    - Yes, all
    - Yes, some
    - No
    - Unknown

## Cardiac arrest registry

42. Is out-of-hospital cardiac arrest reported to a registry? (If yes or in some areas answer questions 42-50)
    - Yes, national registry covering all of the country
    - Yes, national registry covering parts of the country
    - Yes, several local registries
    - Yes, one local registry
    - No
    - Unknown
43. Is in-hospital cardiac arrest reported to a registry?
    - Yes, national registry covering all hospitals
    - Yes, national registry covering some hospitals
    - Yes, several local registries
    - Yes, one local registry
    - No
    - Unknown
44. What year was your out-of-hospital cardiac registry established?
    - Four digit number
45. What year was your in-hospital cardiac registry established?
    - Four digit number
46. How are out-of-hospital cardiac arrest registry cases identified by the registry (multiple choice)?
    - Directly reported electronically from EMS
    - Directly reported on paper from EMS
    - Searching through patient records
    - Identified from dispatch data
    - Unknown
47. What are the case criteria for inclusion on your out-of-hospital cardiac arrest registry?
    - Cardiac arrest confirmed by EMS
    - Resuscitation started
    - Unknown
48. Does your out-of-hospital cardiac arrest registry use Utstein definitions?
    - Yes, 2004
    - Yes, 2014
    - Both 2004 and 2014
    - No
    - Unknown

49. Does your out-of-hospital cardiac arrest registry have a quality assurance process to ensure comprehensive data capture and accurate data recording?
    - Yes
    - No
        - If yes, please explain briefly (open text)
50. First time recorded in your dispatch centre for out-of-hospital cardiac arrest
    - Time call received
    - Time call answered
    - Time call received in medical/ambulance dispatch centre
    - Time call answered in medical/ambulance dispatch centre
    - Time first ambulance resource dispatched to OHCA
    - No dispatch time point recorded
    - Other
    - Unknown
51. When calculating response times, what timestamps from the ambulance are used?
    - Time of first ambulance resource arrival on scene (i.e. car stopped)
    - Time of arrival at patient side
    - Other (please explain)
    - Not calculated
52. Outcome variables available in the registry (multiple choice)?
    - Any ROSC
        - All areas
        - Some areas
    - Sustained ROSC
        - All areas
        - Some areas
    - Status on arrival at hospital
        - All areas
        - Some areas
    - Alive at discharge from hospital
        - All areas
        - Some areas
    - Alive at 30 days
        - All areas
        - Some areas
    - Alive at 1 year
        - All areas
        - Some areas
    - CPC mRS or other at discharge
        - All areas
        - Some areas
    - CPC or mRS 3-6 months after discharge
        - All areas
        - Some areas
    - CPC or mRS more than 1 year after discharge

        - All areas
        - Some areas
    - Quality of life (HUI-3, EQ-5D-3L, EQ-5D-5L, SF-36, SF-36v2, RAND or other standardised quality of life questioners)
        - All areas
        - Some areas
    - Self-defined quality of life questions
        - All areas
        - Some areas

53. Have you published any articles relating to epidemiology from your country the last 10 years?
54. Please list first author and publication year of the last 5 articles from your country.
